# Evaluating Elevated CRP Levels as an Infection Marker: Implications for Chemotherapy Cycles in Lung Cancer Patients

**DOI:** 10.7759/cureus.79243

**Published:** 2025-02-18

**Authors:** Emine Sevil Ayaydin Murtezaoglu, Emre Yılmaz, Tevfik Ozlu

**Affiliations:** 1 Chest Diseases Department, Of State Hospital, Trabzon, TUR; 2 Cardiology Department, Giresun University Faculty of Medicine, Giresun, TUR; 3 Chest Diseases Department, Medical Park Karadeniz Hospital, Trabzon, TUR

**Keywords:** chemotherapy, c-reactive protein, infection marker, lung cancer, physician's decision

## Abstract

Aim: Infection is a prevalent cause of mortality and morbidity in patients with lung cancer. Moreover, infection diagnosis is challenging in such patients. C-reactive protein (CRP) is a frequently used marker in the clinical diagnosis of infection. Notwithstanding the foregoing, CRP may already be elevated in cases of malignant neoplasms, and in addition to malignancy, causal factors such as noninfectious infarction, inflammation, injury, drug reactions, and thrombosis may also be frequently associated with elevated CRP in such patients. The present study investigated the situations wherein higher CRP levels could be considered as the manifestation of infection that might hinder chemotherapy procedures and aimed to determine a cutoff value that could predict infection.

Method: This study prospectively included patients with lung cancer and high CRP values. The clinical features of the patients, along with laboratory and radiographic results, were assessed. Considering the final decision of the physician who followed up with the patients, they were divided into two groups: infected and noninfected groups. Intergroup comparisons were performed for clinical, laboratory, and radiological results. The receiver operating characteristic curve analysis was used to review the diagnostic value of serum CRP levels in predicting infection in patients with lung cancer. The CRP cutoff value to be considered for predicting infection was calculated.

Results: At the time of admission, the mean CRP level of the 180 patients included in the study was 4.5 mg/dL. As per the physician's decision, 23 patients (12.8%) were found to have an infection. For the 23 infected patients, the mean CRP value at admission was 13.0 mg/dL, whereas it was 3.36 mg/dL for the 157 patients who were considered to be noninfected. The mean CRP value of the infected patients was significantly higher compared to that of the noninfected patients (*p*<0.001). For the 23 infected patients who were then treated with antibiotics, the mean CRP levels at admission and after treatment were 12.79 mg/dL and 5.4 mg/dL, respectively. There was a significant difference of 7.35 units between the two levels mentioned above (*p*<0.001). The CRP cutoff value at admission to be considered for predicting infection that would hinder chemotherapy in all patients was ≥6.74 mg/dL with a sensitivity, specificity, positive predictive value, and negative predictive value of 91.3%, 86.6%, 50%, and 98.6%, respectively (*p*<0.001).

Conclusion: This study suggested cutoff values to distinguish infection-associated high CRP levels from other causal factors that might induce an increase in CRP levels in patients admitted for chemotherapy administration. The suggested cutoff values can prevent unnecessary delays in chemotherapy cycles by eliminating false considerations of infection in patients with high CRP levels.

## Introduction

Patients with lung cancer are frequently exposed to infection during disease progression, and infection is considered one of the leading causes of morbidity and mortality [1]. It is a challenging task to diagnose infection, especially in patients with lung cancer who are receiving chemotherapy. This is due to the potential presence of symptoms such as weakness, anorexia, cough, sputum, and shortness of breath that can be confused with the clinical signs of infection. Furthermore, pneumonia may be prevalently confused with conditions such as atelectasis, lymphangitic carcinomatosis, infarction, and tumor progression during lung imaging procedures. Moreover, symptoms of fever and leukocytosis may not be remarkable due to the use of chemotherapeutic agents and analgesic drugs.

C-reactive protein (CRP) is a frequently used marker in the diagnosis of infection. Nevertheless, the CRP level is also elevated in almost all malignant neoplasms [2]. Furthermore, in addition to malignancy, causal factors such as noninfectious infarction, inflammation, injury, drug reactions, and thrombosis may frequently be associated with elevated CRP in such patients [3-5].

Routine clinical, laboratory, and radiological methods are used to investigate infection in patients with lung cancer prior to chemotherapy administration. Due to the abovementioned considerations, chemotherapy administration and, therefore, the treatment of the patient are delayed, and disease control becomes difficult because infection cannot be excluded in some cases. In such cases, it will relieve the clinician and prevent the patient's chemotherapy cycle from being interrupted if it can be established when, under which situations, and to what extent the elevated CRP indicates an infection that would hinder chemotherapy administration. Therefore, this study, derived from our author's thesis, aimed to investigate in which cases elevated CRP should or should not be associated with infection in this patient population and to determine a cutoff value that could predict the presence of infection.

## Materials and methods

Study design

**Figure 1:**
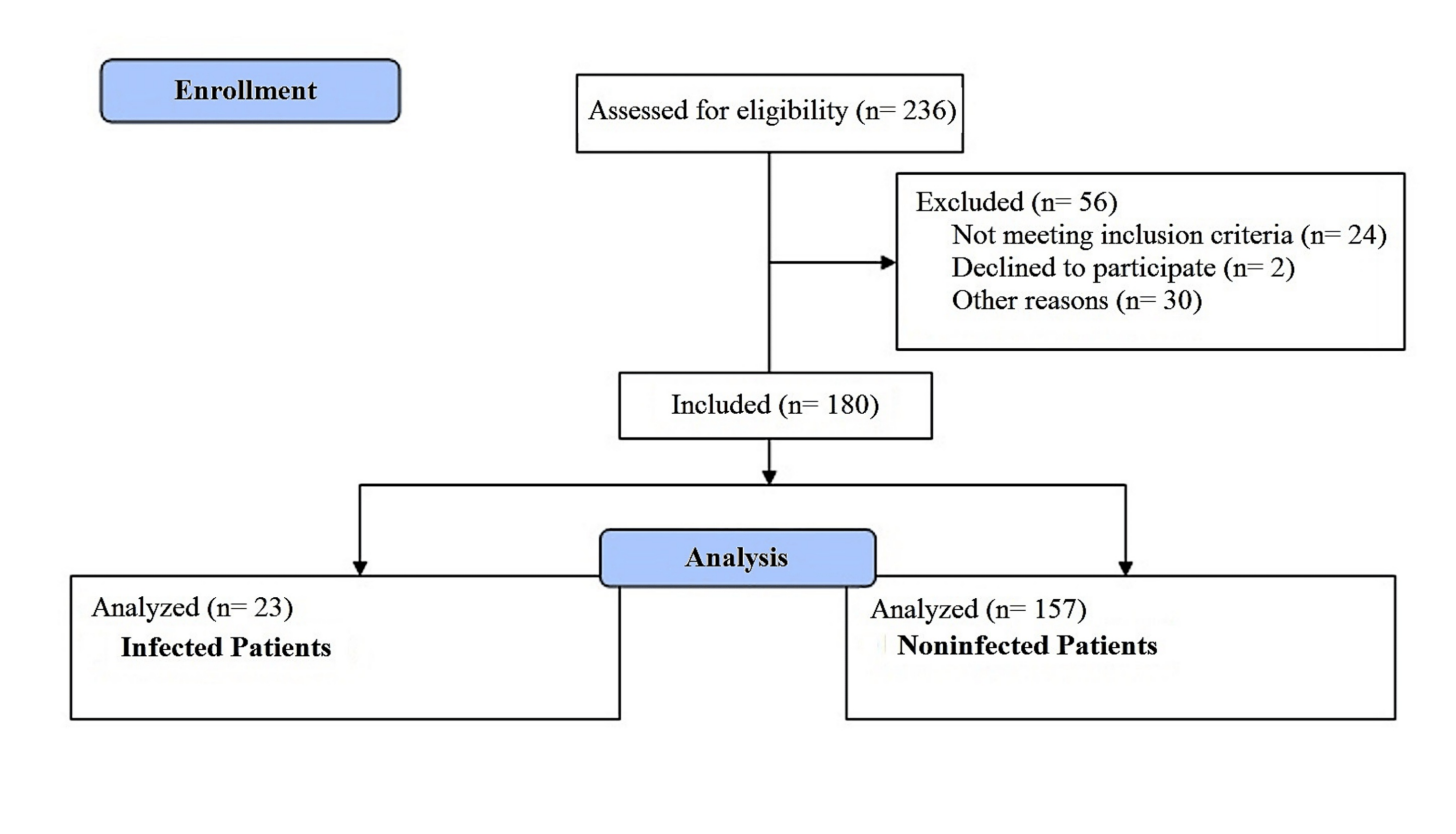
Study flowchart

This was a single-center, prospective, and observational clinical study. According to our inclusion criteria, patients who were diagnosed with lung cancer in our clinic between May 1, 2018, and December 31, 2019, received chemotherapy, had elevated CRP (>0.5 mg/dL), and agreed to participate in the study were included. According to our exclusion criteria, patients with known additional pathology other than lung cancer and those with infection, collagen-vascular disease, secondary malignancy, drug reactions, granulomatous diseases, inflammatory arthritis, familial Mediterranean fever, ulcerative colitis, Crohn's disease, thrombosis, trauma, and infarct that might be associated with elevated CRP levels were excluded from the study (Figure 1).

Etiological examinations (physical examination, blood tests, culture, radiological examinations, etc.) of the cases with high CRP levels, clinical decisions, antibiotic therapy intended for treating the infection, and subsequent chemotherapy results were reviewed. All the demographic and clinical information pertaining to the patients (history of smoking, additional diseases, and use of drugs (nonsteroidal anti-inflammatory drugs, paracetamol, and corticosteroids)); symptoms; physical examination results; cancer types and stages; radiotherapy status; laboratory results (procalcitonin (PCT), sedimentation, polymorphonuclear leukocyte (PNL)/leukocyte ratio, leukocyte count, leukocyturia); radiological infiltration; culture results; CRP levels at admission and before and after the antibiotic therapy; the final decision of the specialist physician; chemotherapy and antibiotic therapy statuses; changes in the laboratory and radiological findings after the antibiotic therapy; whether chemotherapy treatment was administered to the patient at a later date after delayed chemotherapy; and changes in the clinical, laboratory, and radiological findings within the first 10 days after chemotherapy were recorded.

''The final decision of the specialist physician'' was made by a single observer (researcher) in light of objective, radiologic, analytical, measurable, evaluable, and applicable criteria for each patient. These criteria are new radiological infiltration, purulent sputum, leukocytosis, acute-onset fever, new radiological infiltration, signs of infected bronchiectasis on thoracic computed tomography (CT), on physical examination (rales), elevated PCT, upper respiratory tract infection (URTI) findings, dysuria + growth in urine culture, open wound on thighs, marked elevation in CRP levels, and diagnosis of urinary tract infection by an external center. One or more of these criteria led to the decision of an infected patient, especially in the presence of specific symptoms and signs of infection.

In our study, patients were managed with chemotherapy using platinum-based combinations. Cisplatin (carboplatin in patients with renal dysfunction) was combined with Toxeter, Navalbine, or Gemzar in non-small-cell lung cancer (NSCLC) patients. The choice of combination is left to the physician who follows the chemotherapy. In small-cell lung cancer (SCLC) cases, cisplatin was combined with etoposide. The carboplatin + etoposide combination was used in patients with impaired renal function or renal side effects during chemotherapy.

Measurement of serum CRP levels

For determining the CRP levels, we used a Beckman Coulter AU-5800 Autoanalyzer (Brea, California, USA) and the immunoturbidimetric method in which human CRP was combined with latex particles coated with monoclonal CRP antibodies, and the amount of resulting precipitate was calculated. The reference value for CRP was determined to be <0.5 mg/dL. The measurements were performed at the biochemistry laboratory of the Karadeniz Technical University's Faculty of Medicine. Three data were used to analyze CRP level measurements: CRP level measurement at admission before chemotherapy sessions, CRP level measurement at the previous admission, and CRP change (difference between admission CRP level and previous admission CRP level).

Statistical analysis

The IBM SPSS Statistics for Windows, Version 23 (Released 2015; IBM Corp., Armonk, New York) was used in the analyses of data. The Kolmogorov-Smirnov test was used to check whether the variables conformed to a normal distribution. Descriptive variables are expressed as means and standard deviations for the normally distributed variables. A chi-square test was used to compare the infected and noninfected patient groups and investigate whether there was an intergroup difference in terms of gender, symptom presence, examination result, and cancer type. Student's t-test was used to compare the infected and noninfected groups in terms of laboratory values, and a p-value of <0.05 was considered statistically significant. The receiver operating characteristic curve analysis was used to review the diagnostic value of serum CRP levels in predicting infection in patients with lung cancer. The test was considered to have a statistically significant diagnostic value when the Type-1 error level was <5% during the evaluation of the area under the curve. Ethics committee approval was obtained from the Karadeniz Technical University Ethics Committee on Clinical Research (approval no: 2017/132).

## Results

Of the 180 patients included in the study, 171 (95%) were male, and nine (5%) were female. The mean age of the patients was 63.3±7.4 years (minimum: 43 years, maximum: 83 years). Furthermore, 134 patients (74.4%) presented with clinical complaints potentially associated with infection, such as fever in one (0.7%), sputum in 67 (50%), cough in 80 (59.7%), shortness of breath in 75 (56%), URTI in three (2.2%), loss of appetite in 14 (10.4%), dysuria in two (1.5%), and frequent urination in 14 (10.4%). The physical examination revealed that the presenting symptoms were associated with infection in 17 patients (9.4%). Of these 17 patients, 14 (82.4%) had localized rales, two (11.8%) had skin manifestations (hyperemia and elevated temperature), and one (5.9%) had costo-lumbar angle tenderness. Clinical infection manifestations of the 23 patients who were diagnosed with infection as per the final decision of the physician and based on all the clinical, radiological, and laboratory examinations are presented in Table 1.

**Table 1:** Clinical infection manifestations of the 23 patients diagnosed with infection CT: computed tomography, PCT: procalcitonin, URTI: upper respiratory tract infection, CRP: C-reactive protein

Infection Manifestations	Number of Patients (23)
New radiological infiltration + purulent sputum	5
Purulent sputum + leukocytosis	3
Acute-onset fever	2
New radiological infiltration + acute-onset fever	2
New radiological infiltration + leukocytosis	1
New radiological infiltration + purulent sputum + acute-onset fever	1
Purulent sputum + signs of infected bronchiectasis on thoracic CT	1
On physical examination, rales + acute-onset fever	1
Leukocytosis + fever	1
Purulent sputum + elevated PCT	1
URTI findings + elevated PCT	1
Dysuria + growth in urine culture	1
Open wound on thighs + leukocytosis	1
Worsening general condition + marked elevation in CRP levels	1
Diagnosis of urinary tract infection by an external center	1

Information regarding gender, age, lung cancer type and stage, previous chemotherapy history, body temperature, respiratory rate, pulse, blood pressure, leukocyte count at admission, PNL/leukocyte ratio, PCT, erythrocyte sedimentation rate, CRP values of all patients who were determined to be free of infection, and the CRP values during past admission are presented in Table 2. The pulse rate, systolic arterial pressure, leukocyte count, PNL/leukocyte ratio, erythrocyte sedimentation rate, CRP, and PCT values of the infected patients were found to be significantly different compared to those of others.

**Table 2:** Clinical and laboratory features of the patients * Statistically significant p-values (p < 0.05). The p-value is provided for comparisons between the infected and noninfected groups. SD: standard deviation, CRP: C-reactive protein

Clinical and Laboratory Features	All Patients	Infected Patients	Noninfected Patients	p-value
Female/male	9/171	0/23	9/148	0.606
Age (mean±SD)	63.3±7.4	64.12±7.06	63.21±7.54	0.585
Small-cell lung cancer (SCLC)/non-small-cell lung cancer (NSCLC)	43/137	9/14	34/123	0.116
Previously received chemotherapy/chemotherapy-naive	104/76	14/9	90/67	0.924
Stage 1–2/stage 3–4	31/142	1/22	30/120	-
Fever (mean±SD)	36.3±0.4	36.6±0.7	36.2±0.3	0.104
Respiratory rate (per minute ) (mean±SD)	20.4 ± 2.2	21.6±1.9	20.7±2.2	0.140
Pulse ( per minute) (mean±SD)	84.26±11.5	88±21.5	83±10.6	0.024*
Systolic blood pressure (mm/Hg) (mean±SD)	118.7±12.4	114.3±10.7	119.4±12.5	0.039*
Diastolic blood pressure (mm/Hg) (mean±SD)	73.7±7.9	72.6±7.3	73.9±8	0.548
Procalcitonin (mg/dL) (mean±SD)	0.83±4.7	2.3±8.4	0.6±3.8	0.002*
Leukocyte count (mm^3^) (mean±SD)	8714±2942.2	11493±3754.5	8307.3±2576.8	0.001*
PNL/leukocyte ratio (mean ± SD)	64.2±9.8	72.3±7.2	63.1±9.6	0.001*
Erythrocyte sedimentation (mm/hour) (mean ± SD)	56.2±30.2	81.1±25.3	52.6±29.1	0.001*
CRP level at admission (mg/dL) (mean ± SD)	4.5±5.3	13±7	3.36±3.7	0.001*
CRP level at previous admission (mg/dL) (mean ± SD)	3.9±4.6	3.9±3.4	3.9±4.8	0.39

In this study, 137 (76.1%) patients were diagnosed with NSCLC and 43 (23.9%) with SCLC. Of the 180 patients included, 56 (31.1%) had adenocarcinoma, 69 (38.3%) had squamous cell carcinoma, three (1.7%) had large-cell carcinoma, and one (0.6%) had adenosquamous carcinoma. Subtyping was not possible for eight (4.4%) patients. Furthermore, 65 (37.6%) patients had Stage 4B, 23 (13.3%) had Stage 4A, nine (5.2%) had Stage 3C, 23 (13.3%) had Stage 3B, 22 (12.2%) had Stage 3A, 19 (11%) had Stage 2B, six (3.5%) had Stage 2A, and six (3.5%) had Stage 1B cancer according to the Tumor-Node-Metastasis (TNM) classification [6]. In contrast, cancer stage information could not be obtained for seven of 180 participants.

Additionally, 104 (57.8%) patients had previously undergone chemotherapy. The mean admission CRP levels of the patients with Stage 3-4 cancer were higher compared to those of the patients with Stage 1-2 cancer (p=0.005). There was no statistically significant intergroup difference in the admission CRP values between the patients diagnosed with SCLC and those diagnosed with NSCLC or between those with and without prior chemotherapy experience. There was no significant difference between the infected and noninfected groups after 23 infected and 157 noninfected patients were compared in terms of cancer type and stage and prior chemotherapy experience. Physical examination results and new radiological infiltration rates were higher in the infected group compared to the noninfected group (p<0.05).

The mean admission CRP level of 23 patients who were considered as having an infection and administered antibiotic therapy was 12.79 mg/dL. The mean post-treatment CRP level was 5.4 mg/dL (minimum: 0.3 mg/dL, maximum: 23.5 mg/dL) (data of one patient could not be accessed because the patient did not show up after receiving the antibiotic therapy). There was a statistically significant difference of 7.35 units between the admission and post-treatment CRP levels (p<0.0001). Compared to the pretreatment CRP levels, there was a decrease of 57.5% in 14 (63.6%) of 22 patients whose postantibiotic treatment data were available. A chemotherapy cycle was planned for 157 patients without suspected infection, whereas eight patients voluntarily refused the treatment, and the remaining 149 patients (82.8%) received their respective chemotherapy cycles. Of the 149 patients without suspected infection who received chemotherapy treatment, 36 presented to our polyclinic for control purposes within 2-10 days after chemotherapy. One patient had fever + elevated CRP, and another patient had fever + elevated CRP + increased PNL/leukocyte ratio, whereas 34 patients (94.4%) had no symptoms or laboratory results suggesting infection (fever, elevated CRP, increased leukocyte count, increased PNL/leukocyte ratio, or new radiological infiltrate in the lung).

The admission CRP cutoff value for predicting infection that would prevent chemotherapy in all patients was ≥6.74 mg/dL with a sensitivity, specificity, positive predictive value, and negative predictive value of 91.3%, 86.6%, 50%, and 98.6%, respectively (p<0.001). The cutoff values for admission CRP levels that could predict infection in all patients and subgroups and differences between admission CRP and previous CRP levels are given in Table 3. Changes in CRP levels (the difference between admission and previous CRP levels) had a better diagnostic value compared to the admission CRP level alone in predicting infection that would prevent chemotherapy in patients with SCLC and in patients with comorbidities.

**Table 3:** Cutoff values for predicting infection in all patients and specialty groups Sensitivity and specificity values are expressed as percentages (%). AUC: area under the curve, CI: confidence interval, CRP: C-reactive protein, SCLC: small-cell lung cancer, NSCLC: non-small-cell lung cancer

Patient Group	CRP Difference	Cutoff Value	AUC	95% CI	Sensitivity	Specificity
SCLC	CRP at admission (mg/dL)	>7.625	0.865	0.749–0.981	88.9	75
	Increase in the CRP level at admission compared to the previous CRP level (mg/dL)	>5.45	0.869	0.678–1.000	88.9	92.9
NSCLC	CRP at admission (mg/dL)	>6.74	0.861	0.696–1.000	92.9	90.2
	Increase in the CRP level at admission compared to the previous CRP level (mg/dL)	>4.04	0.822	0.681–0.963	100	89.4
≥65 years	CRP at admission (mg/dL)	>6.78	0.909	0.762–1.000	90.9	95.6
	Increase in the CRP level at admission compared to the previous CRP level (mg/dL)	>0.27	0.851	0.703–0.999	88.9	61.9
<65 years	CRP at admission (mg/dL)	>6.74	0.904	0.839–0.970	91.7	79.8
	Increase in the CRP level at admission compared to the previous CRP level (mg/dL)	>5.43	0.848	0.674–1.000	80	93.7
With comorbidities	CRP at admission (mg/dL)	>6.74	0.875	0.752–0.998	92.9	87.8
	Increase in the CRP level at admission compared to the previous CRP level (mg/dL)	>3.74	0.894	0.789–0.998	75	96.6
Without comorbidities	CRP at admission (mg/dL)	>8.73	0.945	0.88–1.000	88.9	91.5
	Increase in the CRP level at admission compared to the previous CRP level (mg/dL)	>5.92	0.784	0.525–1.000	71.4	96.2
All patients	CRP at admission (mg/dL)	≥6.74	0.900	0.820–0.980	91.3	86.6
	Increase in the CRP level at admission compared to the previous CRP level (mg/dL)	≥4.04	0.847	0.729–0.965	73.6	94.3

## Discussion

CRP is a frequently used clinical marker in the diagnosis of infection. CRP secretion from hepatocytes increases due to malignancy; thus, CRP is elevated, especially in almost all patients with malignant tumors [2]. Due to the underlying malignancy, the absolute value of CRP for the evaluation of intercurrent infections has not been fully defined in patients with cancer. In addition to malignancy, causal factors such as noninfectious infarction, inflammation, injury, drug reactions, and thrombosis may frequently be associated with elevated CRP in such patients [3]. Furthermore, it was reported that cytotoxic treatments in these patients slightly increase the CRP levels as a result of tumor destruction [7].

In our study, the mean CRP levels at admission of patients with SCLC and those with NSCLC were 6.06 mg/dL and 4.13 mg/dL, respectively. There was no significant difference in the CRP values at admission of patients with different cancer types. The mean CRP levels at admission of the patients with Stage 3-4 cancer were significantly different compared to those of the patients with Stage 1-2 cancer. A study by Zhao et al. investigating the role of CRP and PCT in differentiating infectious fever and tumor fever in patients with non-neutropenic lung cancer found that CRP levels at the admission of patients with Stage 4 cancer were significantly higher compared to those of patients with Stage 2-3 cancer [8]. In contrast, Gürler et al. found no significant difference between admission CRP levels of patients with advanced cancer and those of patients with early-stage cancer in a study that investigated the effects of chemotherapy on CRP levels and quality of life of patients with cancer [9].

Scheinpflug et al. evaluated 100 patients diagnosed with infectious and noninfectious NSCLC and having elevated CRP levels and found that there was no statistically significant elevation in the infectious NSCLC group compared to the noninfectious NSCLC group [10]. Furthermore, 111 patients were investigated by Schüttrumpf et al. in a study titled "The benefit of PCT level in the evaluation of malignant patients with high CRP plasma concentration," where no statistically significant difference was observed, although the serum CRP concentrations of patients with malignancy and infection were higher compared to those of patients with malignancy and without infection [11]. In a study by Baylak et al. on the serum CRP and PCT levels in patients with NSCLC, the CRP levels of patients with noninfectious NSCLC were found to be significantly higher compared to those of healthy individuals, and the elevation was associated with performance scores [12].

In our study, the comparison of the infected and noninfected groups in terms of laboratory results showed that PCT was significantly higher in the infected group. Additionally, the infected group had a significant elevation of leukocytes, PNL/leukocyte ratio, and erythrocyte sedimentation rate compared to the noninfected group. Although there was no difference between the infected and noninfected hemato-oncology patients in terms of CRP levels, the PCT levels were significantly higher in patients with malignancy and infection in a study by Schüttrump et al. on the significance of PCT in the differential diagnosis of fever of different origins in patients [13]. Moreover, the PCT levels were significantly higher in patients confirmed as having infection in another study by Schüttrump et al. titled "The benefit of PCT level in patients with malignancy and high CRP plasma concentration" that included 111 patients [11]. The results of the relevant literature do not provide a clear perspective regarding the value of CRP levels for predicting infection in patients with cancer. In the present study, there was a statistically significant difference between the infected and noninfected groups in terms of the mean admission CRP value. CRP cutoff values were determined with the aim of predicting an infection that might delay a chemotherapy cycle in patients with lung cancer undergoing chemotherapy. The cutoff value for the CRP level at admission was found to be ≥6.74 mg/dL for predicting the presence of infections that would hinder chemotherapy in all patients.

Furthermore, the cutoff values for the CRP level at admission were >6.74 mg/dL and >8.73 mg/dL for predicting the presence of infections that would hinder chemotherapy in patients with comorbidities and those without comorbidities, respectively. Based on this knowledge, it can be concluded that patients with lung cancer and comorbidities are more prone to infection, and low CRP levels in these patients may act as warning signs of infection.

It was reported that CRP levels were significantly higher in patients with lung cancer compared to healthy individuals [14]. In addition, a study by Palomar-Abril et al. evaluated TNM stage III NSCLC patients by grouping them as under and over 70 years of age, where CRP levels were found to be significantly higher in the group over 70 years of age [15]. In our study, in accordance with the literature, we found that the group older than 65 could provide a higher CRP predictive value for the diagnosis of infection. Unlike the authors, we evaluated both NSCLC and SCLC patients in our study. In addition, we were not limited to Stage 3 patients; approximately 50% of the patients included in our study were Stage 4 patients. Our histopathological heterogeneity and the density of advanced stage patients support the usability of the current literature information in different patients.

In our study, the predictive value of CRP level for infection was higher in the SCLC group compared to the NSCLC group. We can explain this difference as follows: the proportion of SCLC group patients was higher in our infected patient group, and the mean CRP level of the infected patient group was much higher than the noninfected patient group. This distribution of our lung cancer subgroups led to a higher CRP level predictive value for infection in the SCLC group. The higher representation of the SCLC group in the infected group is also consistent with the literature. In a study by Kohno et al. investigating respiratory tract infection patterns in patients with lung cancer, pulmonary infection rates of patients with large-cell lung cancer and SCLC were significantly higher compared to those of patients with NSCLC [16].

The cutoff value for the difference between the admission and previous CRP levels for predicting an infection that would hinder chemotherapy in all the patients was found to be ≥4.04 mg/dL. As suggested in this study, the cutoff values for predicting infection are promising because it is not possible to distinguish infection by definite markers in patients who are admitted, and there are limited studies on this topic.

Compared to the pretreatment CRP levels, 14 (63.6%) of 22 patients whose postantibiotic therapy data were available had a decrease of 57.5%. Similarly, bacteremia was detected in 21 of 96 patients, and 16 (76.2%) of those patients responded to antibacterial treatment in a study by Zhifang et al. investigating the role of CRP and PCT in differentiating infectious and tumor fevers in patients with non-neutropenic lung cancer [8].

In this study, 36 of the 149 patients without suspected infection who received chemotherapy presented to our polyclinic for control purposes within 2-10 days after chemotherapy. No symptom or laboratory result suggested infection in 34 (94.4%) of the admitted patients.

Limitations

This study has certain limitations, including the fact that only the patients with lung cancer were included in the study; therefore, patients with other cancers were not evaluated. The cutoff value for cancer cell types and stages could not be determined due to the limited number of lung cancer cases. No special examination, investigation, treatment, or other medical procedure/intervention was performed on the patients due to the observational design of the study. Hence, data were not available for certain patients.

## Conclusions

In conclusion, CRP cutoff values were determined in the present study with the aim of predicting an infection that might delay chemotherapy in patients with a diagnosis of lung cancer receiving chemotherapy. There was a significant difference between the infected and noninfected groups in terms of the mean admission CRP level. The cutoff value for the admission CRP level was ≥6.74 mg/dL for predicting infections that would hinder chemotherapy in all patients, whereas the cutoff value for the difference between the admission and previous CRP levels for predicting infections that would hinder chemotherapy in all patients was ≥4.04 mg/dL.

## Appendices

Note: This article is derived from the thesis entitled "Should C-reactive protein elevation in lung cancer patients be taken into consideration in postponing chemotherapy cycles?" submitted by Dr. Emine Sevil Ayaydın Mürtezaoğlu to Karadeniz Technical University Faculty of Medicine, Department of Chest Diseases, with the approval of her advisor Prof. Dr. Tevfik Özlü.
